# Severe allergic reactions to the COVID-19 vaccine – statement and practical consequences

**DOI:** 10.5414/ALX02215E

**Published:** 2021-01-05

**Authors:** Jörg Kleine-Tebbe, Ludger Klimek, Eckard Hamelmann, Oliver Pfaar, Christian Taube, Martin Wagenmann, Thomas Werfel, Margitta Worm

**Affiliations:** 1 Allergy and Asthma Center Westend, Berlin,; 2 Center for Rhinology and Allergology of the ENT University Clinic Mannheim, Wiesbaden,; 3 University Clinic for Pediatrics and Adolescent Medicine, Bethel Children’s Center, Evangelical Hospital Bielefeld gGmbH, Bielefeld,; 4 Department of Otorhinolaryngology, Head and Neck Surgery, Section of Rhinology and Allergy, University Hospital Marburg, Philipps-Universität Marburg, Marburg,; 5 Ruhrlandklinik gGmbH, West German Lung Center at the University Hospital Essen – University Hospital,; 6 Clinic of Otorhinolaryngology, University Hospital Düsseldorf,; 7 Clinic of Dermatology, Allergology, and Venerology, Hannover Medical School,and; 8 Allergy Center-Charité, Clinic for Dermatology, Venerology, and Allergology, Campus Charité Mitte, University Medicine Berlin, Germany

**Keywords:** COVID-19 vaccine, BNT162b2, allergic reactions, hypersensitivity, PEG, biologicals, allergen immunotherapy

## Abstract

No abstract available.

On December 10, 2020, the BBC reported two allergic adverse events following the first injection of the BioNTech COVID-19 vaccine BNT162b2. Up to December 19, 2020, severe allergic reactions have occurred in 6 individuals in North America among 272,001 vaccinations. This represents a frequency of ~ 1 : 45,000, well above the expected frequency of a severe allergic vaccine reaction of 1 : 1 million [1]. 

What is the reason behind this? Does this imply an increased risk for allergy sufferers? 

The following statement is a preliminary assessment in light of few cases in countries with available vaccine and current, carefully documented efficacy and safety data from the BioNTech COVID-19 vaccine pivotal clinical trial [2]. 

Apparently, the cases in England involved sufferers with known “severe allergy” who also carried epinephrine auto-injectors for emergencies. The BBC report refers to “anaphylactoid reactions” with possible skin, respiratory, and circulatory involvement. According to CNN, 3 cases in the northern U.S. showed, among other things: a) labored breathing, increased pulse, and a rash on the face and trunk with a biphasic course; b) eyelid edema, dizziness, and itching in the throat; c) tongue swelling, hoarse voice, and labored breathing. 

After epinephrine injection(s), symptoms regressed rapidly in all. Patients a) and c) had no allergic history. 

From an allergists’ perspective, the question arises, what did the affected persons react to? 

As far as is known, the list of ingredients of the vaccines BNT162b2 and mRNA-1273 from Moderna contains, in addition to the modified viral mRNA, carbohydrates, various salts and lipids, one of which is coupled with polyethylene glycol (PEG)-2000. The latter forms so-called PEGylated lipid nanoparticles (LNP) in which the mRNA is embedded and thus stabilized. The non-toxic substance (group) PEG is added to many everyday, cosmetic, and medical products and can trigger systemic allergic reactions [3, 4] in rare cases. Thus, this additive could be a potential cause of IgE-mediated vaccine reactions, as described for other PEG applications [5, 6]. 

On the other hand, PEG, known as a strong stimulus of humoral immune responses, can trigger non-IgE-dependent complement-mediated mast cell activation via IgG or IgM, described as “Complement Activation-Related Pseudoallergy” (CARPA) [7]. In another paper [8], it was shown that IgG and IgM antibodies to PEG were detectable in 7 – 63% of healthy individuals’ serum samples from the 1970s, 1980s, and 1990s. Studies using current sera from healthy individuals (n = 377) show PEG specific antibodies in a mean of 72% of individuals (higher compared to other papers reporting 20 – 30%). 

There are no other PEGylated molecules in other vaccines to date. However, anaphylactoid, non-IgE-mediated clinical reactions have been described against other PEGylated drugs such as liposomal, PEGylated doxorubicin. Whether the observed reactions against Pfizer-BioNTech’s mRNA-based vaccine were triggered by PEG or the LNP is currently still hypothetical. It is now important to carefully monitor current developments, especially incidents during the second vaccinations, and to await further findings. 

An absolute contraindication to COVID-19 vaccinations – as with other vaccines, e.g. against influenza – exists for patients with known hypersensitivity to ingredients of the vaccine. This should rarely be the case for first-time application, given the evidence to date. 

For patients with inhalant allergies, food or insect venom allergies, urticaria or atopic eczema, as well as polyposis nasi or bronchial asthma, vaccines are generally considered safe. The available studies show no evidence for an increased risk of severe allergic reactions to vaccines in general and BioNTech’s COVID-19 vaccine [2]. 

Based on current studies and their mechanism of action, there is no evidence to date of adverse interactions with the COVID-19 disease and the new vaccines for the approved biologics (benralizumab, dupilumab, mepolizumab, omalizumab, reslizumab) for the treatment of atopic and/or eosinophilic airway diseases with type 2 inflammation. Therefore, therapies should be continued as planned and do not need to be interrupted. As generally recommended for inactivated vaccines, vaccinations should be placed approximately midway through the treatment interval, i.e., between two applications of the respective biologics [9]. 

For patients receiving allergen immunotherapy injections (SCIT), the manufacturer’s recommendations shall be considered, i.e., a 1 – 2 weeks interval between SCIT and vaccination. Sublingual immunotherapy (SLIT) can be continued as usual. When using biologics and products for AIT, technical information and directions for use should always be followed. 

Additional recommendations have been provided for download by the Paul-Ehrlich-Institut (German Federal Institute for Vaccines and Biomedines) on December 23, 2020 [10] and a joined statement will be published soon by German allergy societies AeDA, DGAKI, and GPA [11]. As this text was finalized on December 23, 2020, more recent events concerning the new vaccine could not be considered. 

## Funding 

None. 

## Conflict of interest 

None. 
